# Performance and Effectiveness of DeepSeek in Assisting Diagnosis of Acutely and Critically Ill Patients: A Single‐Center Retrospective Study

**DOI:** 10.1002/mco2.70751

**Published:** 2026-04-23

**Authors:** Teng Huang, Jinzhao Zhang, Junpeng Tang, Pengfei Wang, Fanrong Lin, Kaidi Tao, Siqi Liu, Zhengfei Yang

**Affiliations:** ^1^ Department of Intensive Care Unit Sun Yat‐sen Memorial Hospital Sun Yat‐sen University Guangzhou China; ^2^ Department of Emergency Sun Yat‐sen Memorial Hospital Sun Yat‐sen University Guangzhou China

1

Dear Editor:

Critically ill patients often present with nonspecific symptoms and may deteriorate rapidly, creating major diagnostic challenges. Diagnostic errors in emergency and intensive care settings remain substantial and can delay treatment and worsen outcomes. Large language models (LLMs) show promise, but their value in practice requires rigorous evaluation [1, 2]. DeepSeek is a recently developed open‐source LLM with strong reasoning capabilities and local deployment, which protects patient privacy and allows adaptation to regional medical knowledge [3, 4]. Previous LLMs have also provided reliable information for patient education and assisted with patient care [4]. We therefore evaluated DeepSeek's performance in assisting intern physicians with admission diagnoses of critically ill patients.

We performed a single‐center, retrospective evaluation of 200 adults, representing all consecutive eligible critically ill admissions, admitted to the emergency intensive care unit (EICU) at Sun Yat‐sen Memorial Hospital between January and December 2023. The study was approved by the institutional ethics committee, with waiver of individual consent. Because the cohort was defined by this fixed 1‐year time window, no a priori sample size calculation was performed. Admission data (history, examination, initial laboratory tests, and imaging) were assessed using three approaches: (i) Intern‐only: three interns with 2–3 years of clinical experience provided independent diagnoses; (ii) DeepSeek‐only: DeepSeek generated diagnoses from the same data; and (iii) Assisted‐intern: interns first diagnosed independently, then reviewed DeepSeek's output before finalizing their diagnosis. Interns received brief training on interpreting AI outputs.

We used DeepSeek‐R1, a reasoning model released in January 2025, with 671B parameters. Search and deep‐think features were disabled. The model ran in fresh sessions with no prior user association or cross‐case memory. The same prompt was used for all cases: act as a board‐certified physician with expertise in clinical reasoning, differential diagnosis, and evidence‐based treatment protocols. Your task is to analyze patient medical records and produce an exhaustive diagnostic evaluation followed by a tailored management plan, prioritizing completeness, safety, and alignment with current guidelines.

Three senior chief physicians, each with more than 10 years of EICU experience, independently reviewed each case while blinded to study arms. Disagreements were resolved through a Delphi consensus to establish the reference diagnosis. The primary outcome was diagnostic accuracy, defined as agreement with the reference primary diagnosis. Secondary outcomes were time to diagnosis and a 12‑point diagnostic score that rewarded a correct primary diagnosis (6 points), relevant additional diagnoses (4 points), and concise reasoning (2 points). Accuracies were compared using chi‐square tests. Because times were not normally distributed, we used Kruskal–Wallis tests for time and one‐way ANOVA with Bonferroni adjustment for scores. Two‐sided *p* values < 0.01 were considered significant.

Among the 200 critically ill patients, 118 were male, and 82 were female. The mean age was 58.4 ± 16.7 years. The main reasons for admission included severe infectious diseases, acute cardiovascular events, major trauma and bleeding, critical neurological conditions, and other severe medical and surgical conditions. Among the 200 patients, interns alone correctly diagnosed 136 cases, with an accuracy of 68.0%. DeepSeek alone correctly diagnosed 172 cases, with an accuracy of 86.0%. The interns assisted by DeepSeek correctly diagnosed 158 cases, achieving an accuracy of 79.0%. Differences in diagnostic accuracy among the three groups were statistically significant (*p* < 0.01). Post hoc pairwise comparisons showed that DeepSeek, either alone or assisting interns, yielded statistically significant improvements in diagnostic accuracy compared with interns alone (both *p* < 0.01). Introducing DeepSeek assistance increased diagnostic accuracy for interns by 11% (95% CI, 4.0%–18.0%).

To further assess diagnostic quality, we analyzed diagnostic scores. The mean diagnostic scores were 8.7 ± 1.0 for interns alone, 9.9 ± 0.6 for DeepSeek alone, and 9.6 ± 0.8 for interns assisted by DeepSeek. The differences among the groups were statistically significant (*p* < 0.001). This indicates that DeepSeek, either independently or as an assistant, provided more comprehensive and accurate diagnostic insights compared to interns alone. Diagnosis time also differed between groups. Interns alone took an average of 11.3 ± 4.5 min per patient. DeepSeek alone provided the fastest diagnoses, averaging only 2.1 ± 1.0 min per case. The intern‐plus‐DeepSeek group had an intermediate average diagnosis time of 7.0 ± 3.2 min. Compared with interns diagnosing independently, the interns assisted by DeepSeek statistically significantly reduced the time spent on clinical reasoning and information retrieval. Analysis showed statistically significant differences in diagnosis time among the three groups (Kruskal–Wallis *H* = 86.5, *p* < 0.01). Compared to the intern group, the intern‐plus‐DeepSeek group reduced diagnosis time by 4.30 min on average (95% CI, 4.06–4.54, *p*<0.001). This corresponds to approximately a 38% reduction in diagnosis time (Figure 1).

**FIGURE 1:**
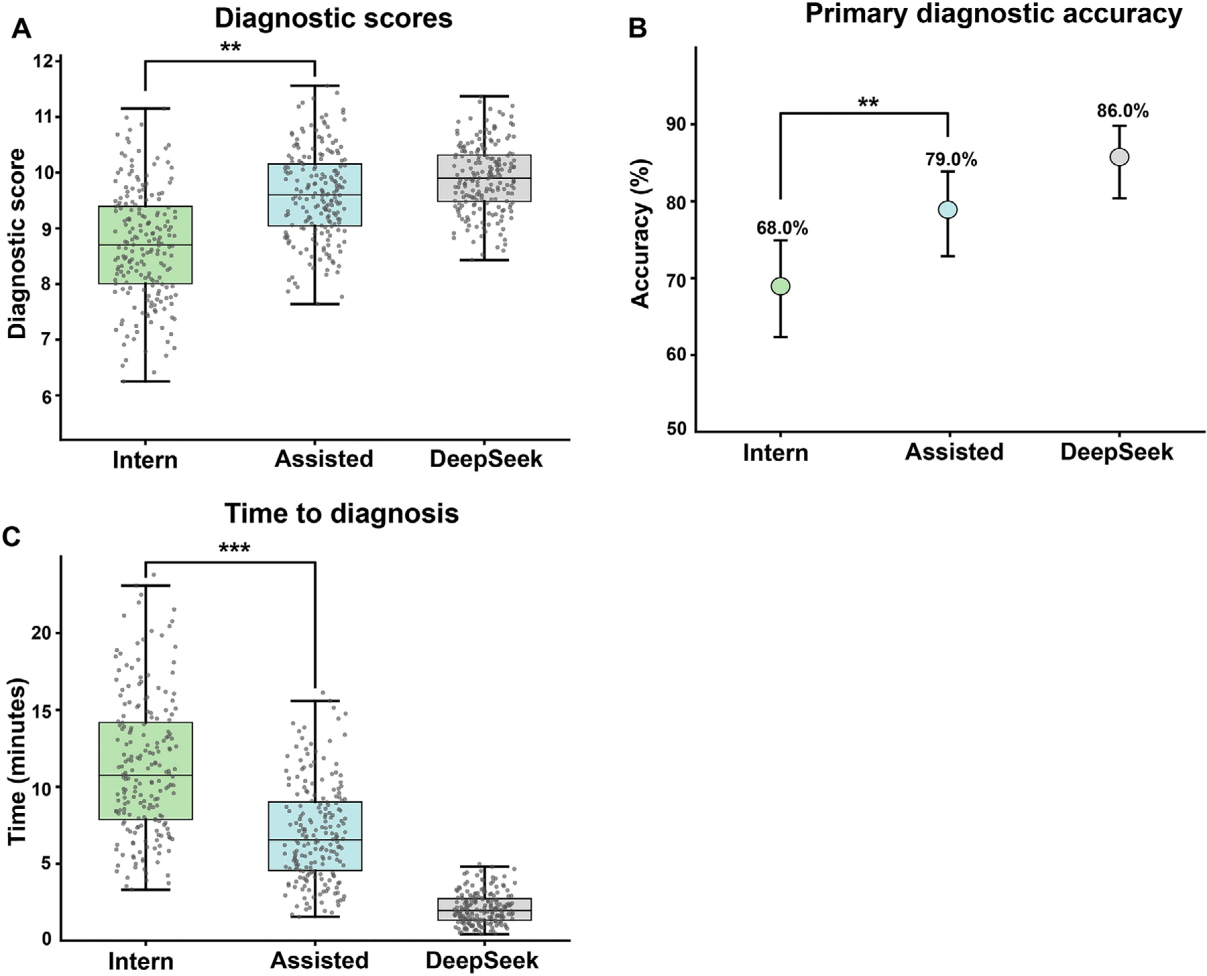
(A) Distribution of diagnostic scores shown as box plots with overlaid individual data points. (B) Primary diagnostic accuracy shown as point estimates with 95% confidence intervals. (C) Distribution of diagnosis time (minutes) shown as box plots with overlaid individual data points.

The most important finding of this single‐center study is that DeepSeek assistance improved interns’ diagnostic accuracy and efficiency at EICU admission, while DeepSeek alone delivered faster performance and higher accuracy. These results suggest practical value for junior clinicians working under time pressure. For implementation and potential bias, real‐world deployment will require standardized inputs, clear governance of prompts and versioning, mechanisms that surface uncertainty, and safeguards against automation bias. Human supervision should remain mandatory at every step, particularly when LLM suggestions influence acute care decisions [1, 5]. As an open‐source model, DeepSeek may also lower licensing costs compared with closed, subscription‐based LLMs.

This study has several limitations. First, as it was conducted at a single center, the generalizability of DeepSeek's performance to other clinical settings and broader patient populations requires further validation. Second, although the sample size of 200 cases is statistically adequate, it may still be insufficient to thoroughly evaluate the diagnostic capability of AI for certain rare conditions. Third, the diagnostic process in the intern‐plus‐DeepSeek group involved an open, interactive approach, potentially causing variability in the extent to which interns relied on AI recommendations and introducing subjectivity. Finally, our evaluation focused primarily on diagnostic accuracy rather than patient outcomes. While improved diagnostic accuracy should logically enhance clinical treatment decisions, direct evidence of improved patient outcomes needs confirmation through subsequent randomized controlled trials.

In summary, DeepSeek demonstrates high standalone diagnostic accuracy and, as an assistive tool, enhances interns’ diagnostic performance in critical care. These findings support careful, supervised integration of LLMs to reduce diagnostic error and speed early decision‐making.

## Author Contributions

T. Huang and J. Zhang conceptualized and designed the study. J. Tang, P. Wang, F. Lin, and K. Tao contributed to data collection, data curation, and verification. T. Huang and J. Zhang performed the statistical analyses and drafted the first version of the manuscript. S. Liu and Z. Yang provided clinical oversight, interpreted the findings, and critically revised the manuscript for important intellectual content. All authors have accepted responsibility for the entire content of this manuscript and approved its submission.

## Funding

This research was supported by grants from the National Natural Science Foundation of China (No. 82372207).

## Ethics Statement

All procedures performed in this study were in accordance with the ethical standards of the Ethics Committee of Sun Yat‐sen Memorial Hospital (Approval No.: SYSKY‐2024‐1131‐01).

## Conflicts of Interest

The authors declare no conflicts of interest.

## Data Availability

The data that support the findings of this study are available from the corresponding author upon reasonable request.
